# Targeting RNA Structure to Inhibit Editing in Trypanosomes

**DOI:** 10.3390/ijms241210110

**Published:** 2023-06-14

**Authors:** Francis A. Acquah, Blaine H. M. Mooers

**Affiliations:** 1 Department of Biochemistry and Molecular Biology, University of Oklahoma Health Sciences Center, Oklahoma City, OK 73104, USA; francis.asieduacquah@gmail.com; 2 Stephenson Cancer Center, University of Oklahoma Health Sciences Center, Oklahoma City, OK 73104, USA; 3 Laboratory of Biomolecular Structure and Function, University of Oklahoma Health Sciences Center, Oklahoma City, OK 73104, USA

**Keywords:** RNA–drug interactions, virtual screening, RNA–ligand interactions, RNA microscale thermophoresis, computer-aided drug design, RNA drug discovery, RNA targets, trypanosome RNA editing, small molecule–RNA docking, unsupervised machine learning

## Abstract

Mitochondrial RNA editing in trypanosomes represents an attractive target for developing safer and more efficient drugs for treating infections with trypanosomes because this RNA editing pathway is not found in humans. Other workers have targeted several enzymes in this editing system, but not the RNA. Here, we target a universal domain of the RNA editing substrate, which is the U-helix formed between the oligo-U tail of the guide RNA and the target mRNA. We selected a part of the U-helix that is rich in G-U wobble base pairs as the target site for the virtual screening of 262,000 compounds. After chemoinformatic filtering of the top 5000 leads, we subjected 50 representative complexes to 50 nanoseconds of molecular dynamics simulations. We identified 15 compounds that retained stable interactions in the deep groove of the U-helix. The microscale thermophoresis binding experiments on these five compounds show low-micromolar to nanomolar binding affinities. The UV melting studies show an increase in the melting temperatures of the U-helix upon binding by each compound. These five compounds can serve as leads for drug development and as research tools to probe the role of the RNA structure in trypanosomal RNA editing.

## 1. Introduction

Infections caused by trypanosomatid pathogens threaten the health of more than a billion people worldwide and often result in long-term disfiguring disabilities or death [1,2]. Current drugs have poor efficacy, adverse side effects, and complex treatment and administration protocols [3]. Furthermore, the parasites exhibit growing resistance to the current drugs [4]. Thus, there is an unmet need to develop safer and more effective drugs to tackle these diseases. The extensive editing of mitochondrial mRNA transcripts in trypanosomes is essential for the survival of the parasite, but a similar mitochondrial RNA editing system is absent in humans [5]. This mRNA editing occurs after transcription and involves the insertion, deletion, or both of the uridylates in the pre-edited mitochondria mRNA (pre-mRNA). Large ribonucleoprotein complexes, known as editing complexes, perform the edits under the direction of many guide RNAs (gRNAs), which provide templates for controlling the editing reactions [6].

This editing process has been an attractive drug target with numerous pharmaceuticals directed at inhibiting the protein components of the editosome [7,8]. However, there is little to no information regarding the discovery of inhibitors that target the heterodimer formed by the guide RNA and the segment of the mRNA that is the target of the editing reactions. The gRNAs are composed of the following three functional domains: a 5′ anchor sequence that finds the editing site by forming complementary base pairs at the anchor binding site (ABS) in the pre-mRNA (anchor helix); a template domain that binds to the editing site through the Watson–Crick and non-Watson–Crick base pairing; and a 3′ oligoU tail that binds to the pre-mRNA upstream of the editing site to form a double helix (the U-helix) [9,10]. Structural studies on the U-helix of the duplexes revealed features and conformations that can be targeted with high-affinity small-molecule binders to either prevent efficient pairing or induce stabilization to inhibit subsequent editing reactions [11,12,13].

Here, we test the hypothesis that the U-helix may act as a target for small-molecule compounds. These compounds may bind and disrupt the recognition of the editing site by the editing complexes or induce duplex stabilization that suppresses subsequent editing reactions. We used virtual screening and molecular dynamics simulations to identify high-affinity binders to the U-helix. We experimentally validated the lead compounds using binding and melting assays. We characterized the conformational changes in the U-helix upon ligand binding by using circular dichroism. Our results identify new compounds selective for the gRNA–pre-mRNA editing substrate that can be developed into editing inhibitors and trypanocidal compounds. Our results will be of particular interest to the field of RNA drug design because it highlights some of the essential steps required for hit discovery and the development of RNA-targeted therapeutics.

## 2. Results

To discover a set of lead compounds that bind to the major groove of the U-helix, we used a combination of computational and experimental approaches. We screened a large chemical library on the computer and extracted a diverse subset of compounds from the top percentile for further analysis. The selected compounds were subjected to molecular dynamics simulations to determine if the drug remained bound to the RNA during a 50-nanosecond simulation. The compounds that passed this validation step were then subjected to experimental testing of their binding to the U-helix RNA and a control RNA composed of all Watson–Crick base pairs. The binding affinity was determined via microscale thermophoresis, and the impact of the compound binding on the RNA conformation was determined via CD spectroscopy.

### 2.1. Hit Identification by Virtual Screening

We targeted a site in the major groove of the U-helix with three adjacent GU wobble base pairs that were associated with the local opening of the major groove as the center of the box used in the virtual screening. The major groove was selected due to its richer array of H-bond donors and acceptors [14]. We used a 1.05 Å crystal structure of the U-helix as the target structure (PDB-code 5DA6). We virtually screened 262,000 compounds selected from the ZINC15 database [15] and from the Diversity Set of the DTP program of the NIH/NCI. We used AutoDock Vina version 1.1.2 and a supercomputer to simulate the docking [16,17].

**Figure 1:**
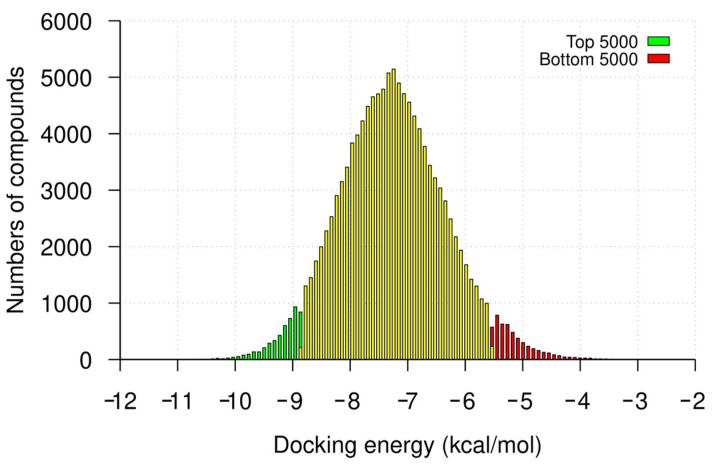
Frequency distribution of virtual screening docking energies. The counts of the top- and bottom-ranked 5000 compounds from the virtual screening are highlighted in green and red, respectively. The counts of the docking scores for the remaining compounds are highlighted in yellow. The histogram is divided into bins using a bin width of 0.1 kcal/mol.

Following the virtual screening of the library of compounds, we ranked the compounds by docking the energies toward the 32 nt U-helix. The docking energies for the top 5000 compounds ranged from −14.6 to −8.8 kcal/mol (mean ± SD: −9.2 ± 0.3 kcal/mol), while those for the bottom 5000 compounds ranged from −5.5 to −2.6 kcal/mol with an average of −5.13 ± 0.4 kcal/mol (Figure 1). We used a Welch *t*-test to compare the binding energies of the top- and bottom-ranked compounds. This test shows a significant difference between the two groups (*p*-value < 0.0001; Welch-corrected t = −555, df = 8728.6).

**Figure 2:**
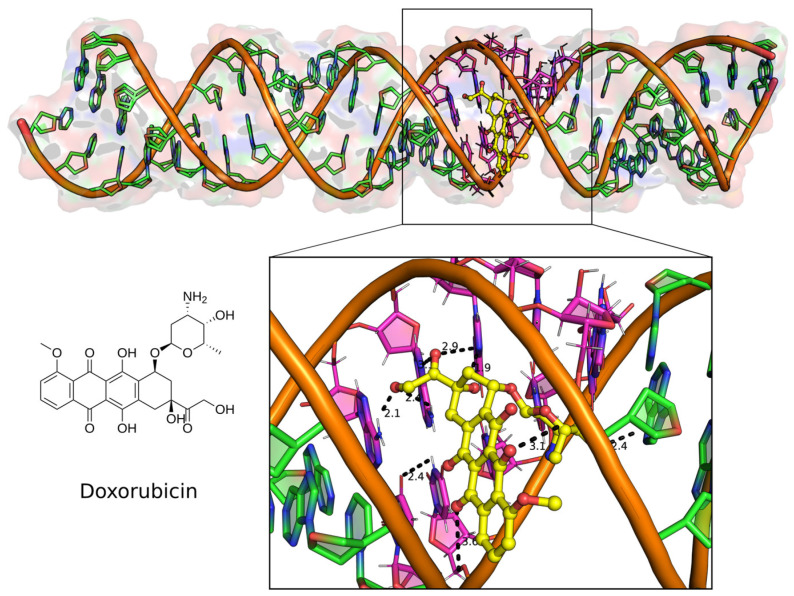
Docked complex of the top binding mode of Doxorubicin (**left**) in the major groove of the 32 bp U-helix (**top**). Major groove binding poses of the drug-making polar contacts to the G-U base pairs in the groove (**below**). Doxorubicin is shown as yellow ball and stick model. RNA helix is shown as filled ring cartoon with the G-U base pairs colored magenta. Polar contacts are shown as black broken lines with distance labels indicated in Angstroms.

For comparison, neomycin and tetracycline, two known RNA binders, had docking energies of −9.3 and −9.5 kcal/mol, respectively. Structural analysis of some of the compounds in the major groove showed that the compounds fit well. Most of the forces mediating groove interactions were hydrogen-bonding contacts between the uracil N2 and guanine O6 atoms and the OH groups of the compounds. The three contiguous G-U wobble base pairs were involved in maintaining interactions in the major groove (Figure 2).

### 2.2. Chemoinformatic Analysis

**Figure 3:**
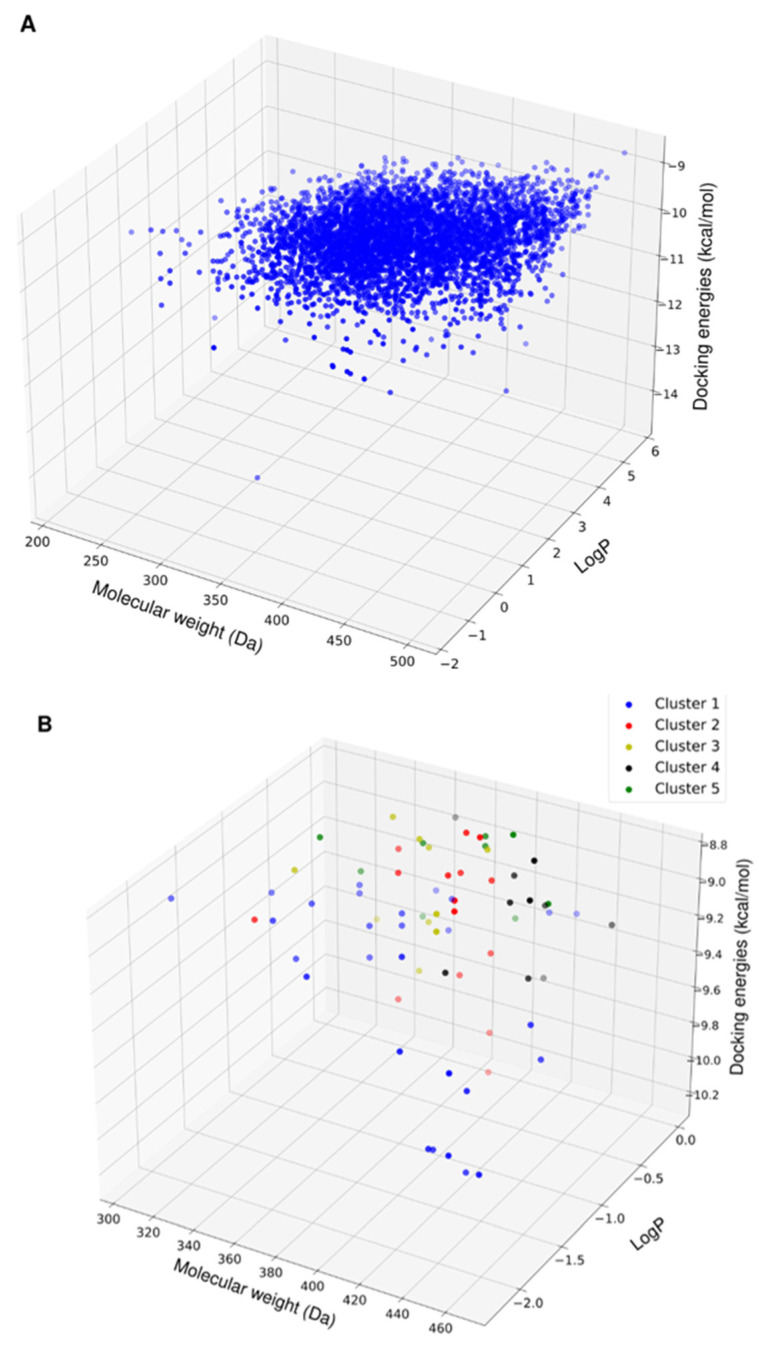
3D scatter plot of the physicochemical properties of the top hits from virtual screening. (**A**) Physicochemical properties of the top 5000 compounds as ranked by docking energies. (**B**) Physicochemical properties of the reduced set of compounds with favorable aqueous solubility color-coded by their clusters based on molecular fingerprint similarity.

Following the virtual screening, the physicochemical properties of the top 5000 compounds showed a molecular weight range of 200–450 Da and a *LogP* range from −1 to 6 (Figure 3A). To avoid solubility issues downstream, we removed the compounds with positive *LogP* values. This purge left 365 compounds (Figure 3B).

Screening all of these compounds via MD simulation was too expensive, so we applied a different approach to the compound selection. Rather than performing MD simulations on only the top 20 compounds, we selected hits that were diverse in their chemical structure and physiochemical properties by using the Butina clustering algorithm with a Tainamoto similarity cutoff of 0.7 [18]. This cutoff allowed for the generation of diverse clusters of compounds, while each cluster had similar compounds. The algorithm provided 14 clusters with five or more compounds. The molecular weight, solubility properties, and docking energies of the members of the clusters did not show distinct patterns of grouping or segregation (Figure 3B). This result implies that the molecular fingerprint clustering approach was unbiased toward a limited range of compounds.

### 2.3. Molecular Dynamic Simulations

Based on the clustering results, we selected representative compounds from the top 50 clusters and other compounds that we had available for the MD simulations. Our motivation for performing the MD simulations as a post-docking validation step was to inform the selection of candidates’ compounds for ligand binding experiments.

**Figure 4:**
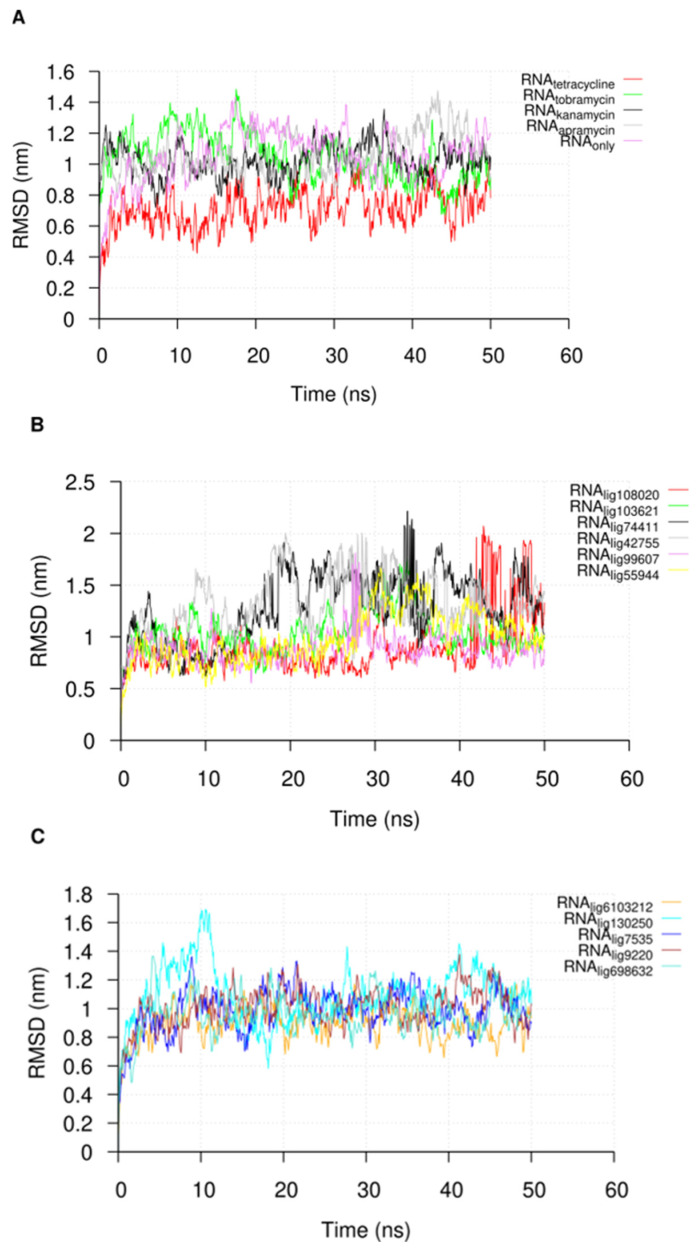
The root-mean-square deviation (RMSD) plot of the nucleotide atoms in the U-helix when in complex with (**A**) known RNA binders and (**B**,**C**) different compounds during the molecular dynamics simulation. The name of the compound is in the subscript in the key label. The trajectories were sampled every 50 ps.

**Figure 5:**
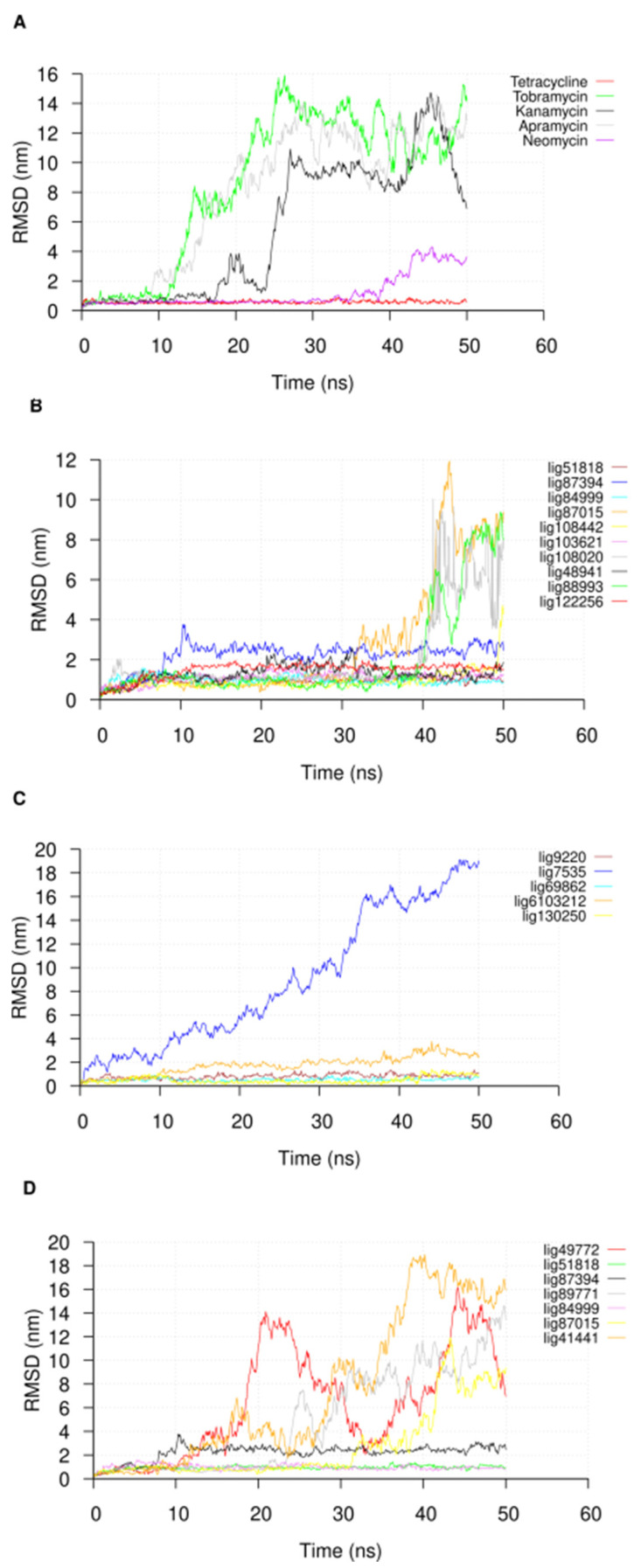
The RMSD plot of (**A**) known RNA binders and (**B**–**D**) different compounds used in the 50 ns molecular dynamics simulations. The trajectories were sampled every 50 ps.

Following the 50 ns of MD simulation, we extracted the U-helix RNA RMSDs from the simulation trajectories to check for the convergence of the simulation. The RMSDs of the compounds in the major groove starting position were also extracted to check their stabilities. An analysis of the U-helix RMSD shows a convergence of the system toward an equilibrated state with relatively low RMSD values (Figure 4). The small molecule RMSD analysis in complex with the U-helix showed equilibration for 15 compounds that formed stable complexes for at least 10 ns of the simulation (Figure 5A,B). The remainder of the compounds moved out of the major groove and into the bulk solvent, as seen in a sharp increase in the average RMSD values (Figure 5C,D).

**Figure 6:**
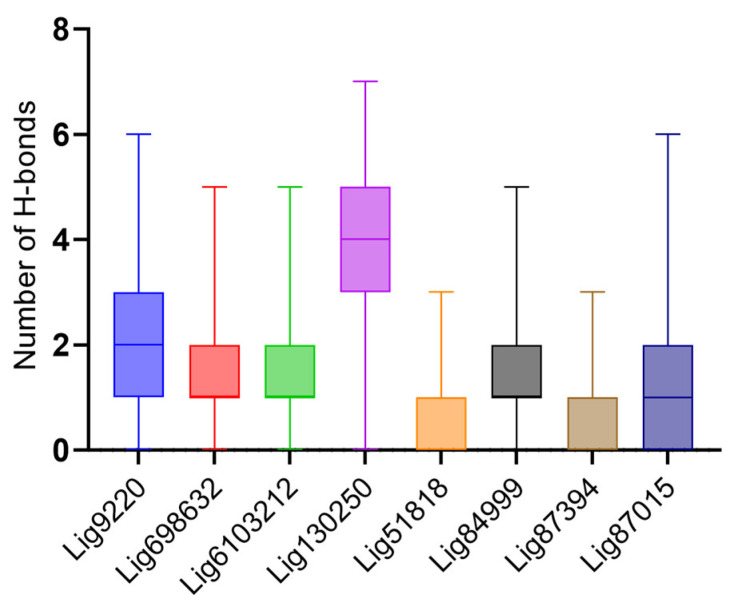
Hydrogen bonding description between the U-helix and the different compounds during simulations. Box plot shows the distribution of the number of hydrogen bonds formed by the top eight compounds showing low RMSD values.

We analyzed the hydrogen bonding between the various compounds with low RMSDs and the U-helix to better understand the forces mediating the interactions. While the majority of the low RMSD compounds formed one or two hydrogen bonds, three compounds (Lig9220, Lig130250, and Lig87015) formed up to six hydrogen bonds with the U-helix throughout the simulation (Figure 6).

**Figure 7:**
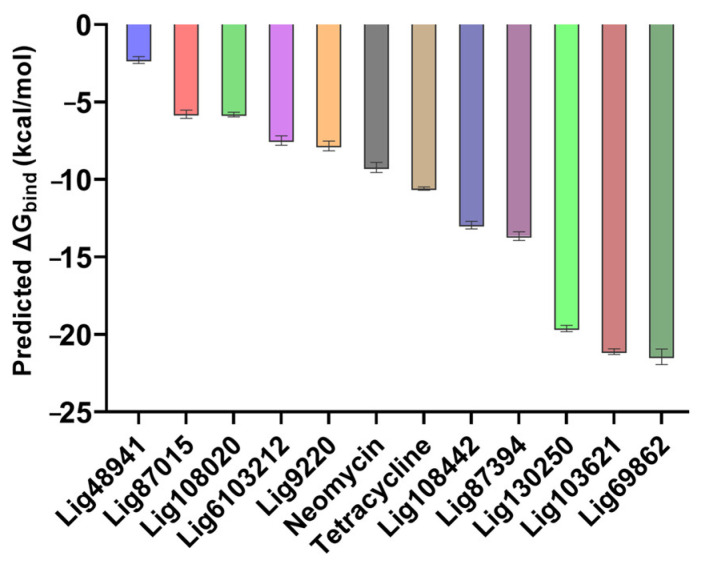
Predicted binding free energies of compounds computed during the 50 ns simulations. The known RNA-binding compounds are added for comparison. Data for predicted binding energy are shown as mean +/− SEM; *n* = 1000.

As seen from the energetics of the simulation, the predicted binding energies from the MMGBSA calculations were consistent with the previous results because the compounds with low average RMSD values also had more favorable predicted binding energies and vice versa (Figure 7).

### 2.4. Top Hit Compounds Show Direct Binding to U-Helix with High Affinities

**Figure 8:**
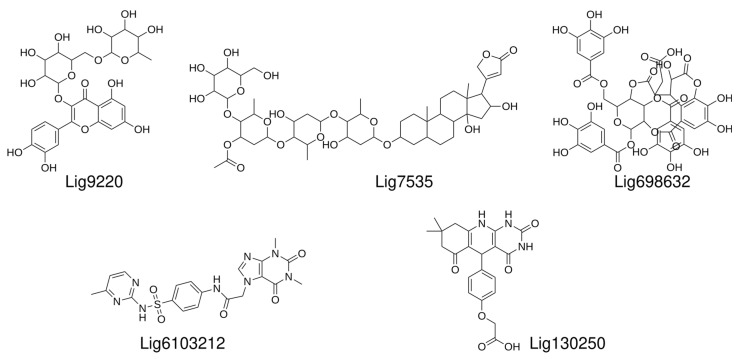
Chemical structures of hit compounds with measured affinities toward labeled U-helix.

Based on the above MD simulations and predicted binding energies, we settled on nine compounds for the in vitro validation via in vitro ligand binding experiments. The initial binding experiments did not show detectable binding affinities to the labeled U-helix for four of the nine compounds and were dropped from further analysis. The chemical structures of the remaining five compounds that demonstrated binding to the labeled U-helix are shown in Figure 8.

**Figure 9:**
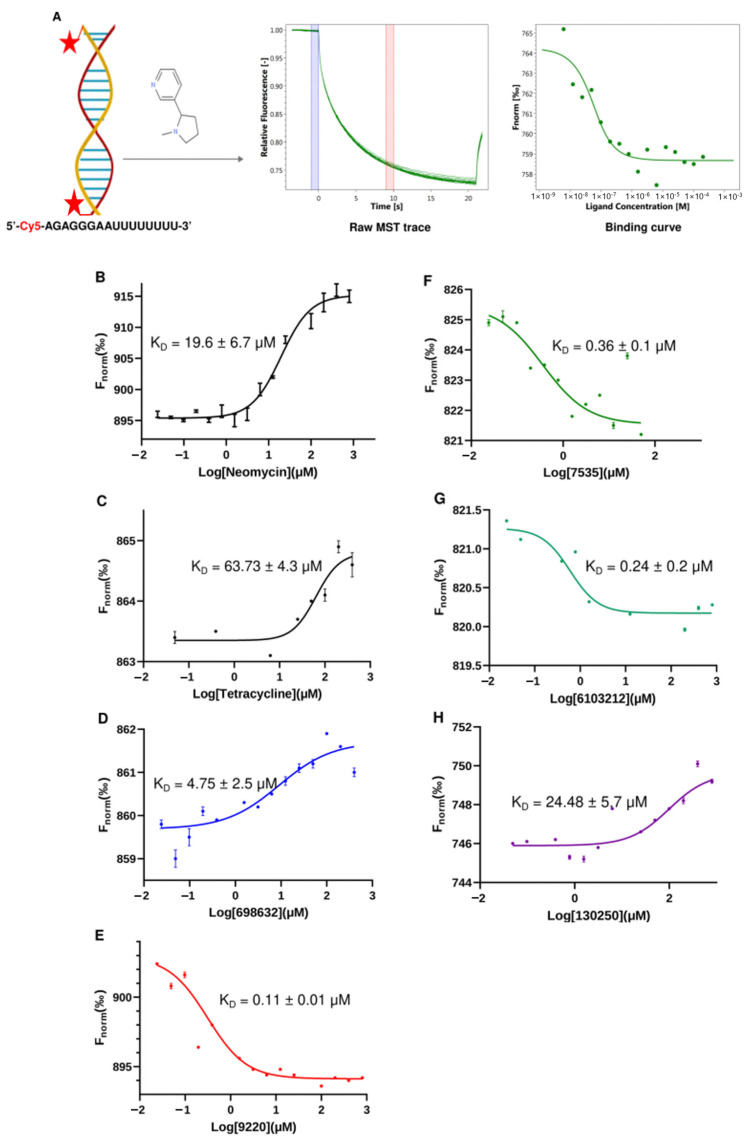
Development of MST binding assay to test hit compounds in vitro. (**A**) Cartoon schematic of the microscale thermophoresis assay. The red stars denote Cy5 (Sulfo-Cyanine5), which is the fluorescent dye covalently attached to the 5′ end of each RNA strand. Purple and pink shaded areas in the middle figure denote F_cold_ and F_hot_ areas respectively. Average fluorescence is calculated in each area and the change in normalised fluorescence (ΔFnorm) used in plotting binding curve is defined as F_hot_/F_cold_. Binding curves generated via MST analysis for the binding of labeled U-helix to (**B**,**C**) neomycin and tetracycline (positive controls) and (**D**–**H**) compounds Lig6986832, Lig9220, Lig7535, Lig6103212, and Lig130250. Data points represent the means of triplicate experiments. Error bars represent the standard error of the mean (SEM). When not visible, the error bars are smaller than the symbols.

We selected MST to measure the direct binding of the compounds to the U-helix for several reasons. MST requires less material than isothermal titration calorimetry (ITC) and avoids the immobilization issues of surface plasmon resonance (SPR). Additionally, MST can detect binding affinities in the nanomolar and sub-nanomolar concentration ranges [19]. As shown in Figure 9, we developed a simple MST assay using a 16 bp version of the U-helix labeled at the 5′ end with Cy5. A representative MST trace shows that the labeling did not affect the small molecule binding (Figure 9A). Furthermore, a distinct change in the thermophoretic behavior between the bound and unbound U-helix allowed for the accurate estimation of the binding affinity from a single binding event. The two known RNA-binding compounds (neomycin and tetracycline) exhibited a K_D_ of 19.66 µM and 63.73 µM, respectively. The five hit compounds gave K_D_s values ranging from 0.11 µM to 24.48 µM to the labeled U-helix. Compared to the standard compounds, these compounds demonstrated favorable micromolar affinities to the RNA editing substrates comparable to the earlier reported values [20]. The labeled U-helix had a negative thermophoretic behavior for the compounds Lig9220, Lig7535, and Lig6103212. This behavior resulted in a change in the shape of the binding curve (Figure 9E–G). This observation might have been due to a change in the RNA conformation upon the ligand binding of these compounds, which does not affect a concentration-dependent thermophoretic behavior.

**Figure 10:**
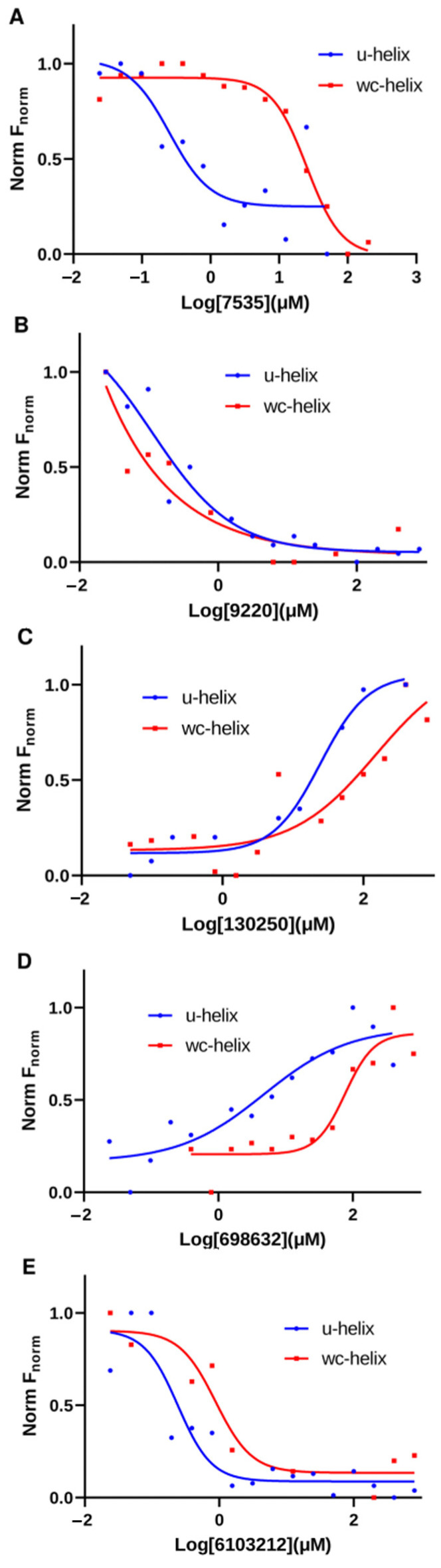
Comparison of binding profiles of compounds between binding to U-helix and W-C helix. MST binding curves of hit compounds to U-helix (blue curve) and to W-C helix (red curve) at same concentration ranges. (**A**) Lig7535, (**B**) Lig9220, (**C**) Lig130250, (**D**) Lig698632, and (**E**) Lig6103212.

We tested the binding of the five compounds against a labeled 16-nucleotide WC-helix (where the G-U base pairs are replaced with G-C base pairs) using our MST binding assays. This control helix was used to eliminate compounds with non-specific RNA-binding behavior from our list. From the binding curves obtained, four out of the five compounds showed a higher affinity to the U-helix (shifted left) compared to the WC-helix (Figure 10). For Lig9220, the fitted curve showed a K_D_ of 0.11 µM for the U-helix and <0.0001 µM for the WC helix. Taking it all together, the binding experiments after the in silico selection narrowed our initial set of compounds to four leads, with micro- and sub-micromolar affinity to the 16 bp U-helix.

### 2.5. Lead Compounds Stabilize U-Helix RNA without Affecting Helical Conformation

**Figure 11:**
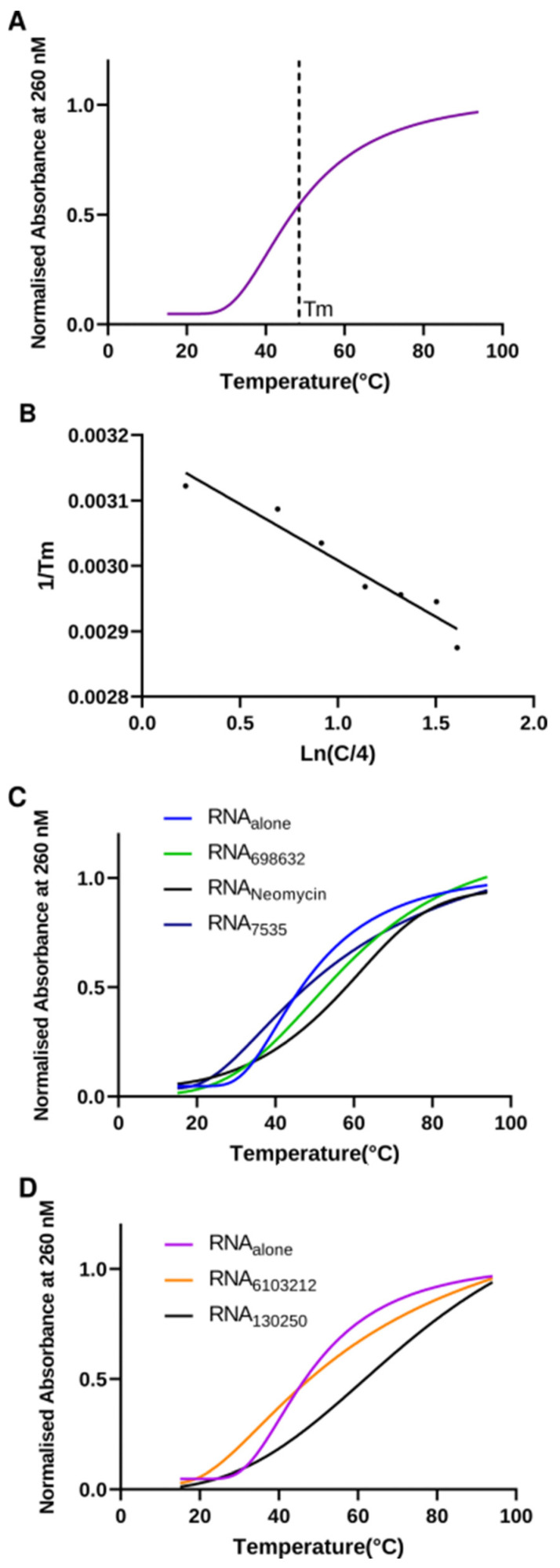
Thermodynamic stability analysis of U-helix RNA upon small molecule binding. (**A**) Representative normalized UV melting curve of 12.5 µM U-helix RNA alone. (**B**) Van’t Hoff plot (1/Tm versus ln(CT/a)). (**C**,**D**) Representative UV melting curves of RNA alone and in complex with various hit compounds at concentrations of 10X the K_D_.

As a follow-up to our binding assay, we investigated the thermodynamics of the U-helix upon hit compound binding. Previous studies have shown thermal stabilization of nucleic acids upon ligand binding [21,22]. Using a UV-melting experiment of the U-helix in a defined compound concentration, we obtained the melting curves, as shown in Figure 11. In the absence of any compound, the U-helix showed a single transition. The melting temperatures showed that the U-helix stability increased upon the binding of the compounds (Figure 11C,D). The subsequent Van’t Hoff plot analyses relating the melting temperatures (T_m_), strand concentration (CT), enthalpy change (ΔH°), and entropy change (ΔS°) were used to extrapolate the enthalpy and entropy change (Table 1). Interestingly, the higher melting temperatures (T_m_) observed in the binding of the Lig6103212 and Lig130250 did not lead to larger enthalpy changes. Previous studies also supported the notion that the melting temperatures may be insensitive to enthalpy changes [23].

**Figure 12:**
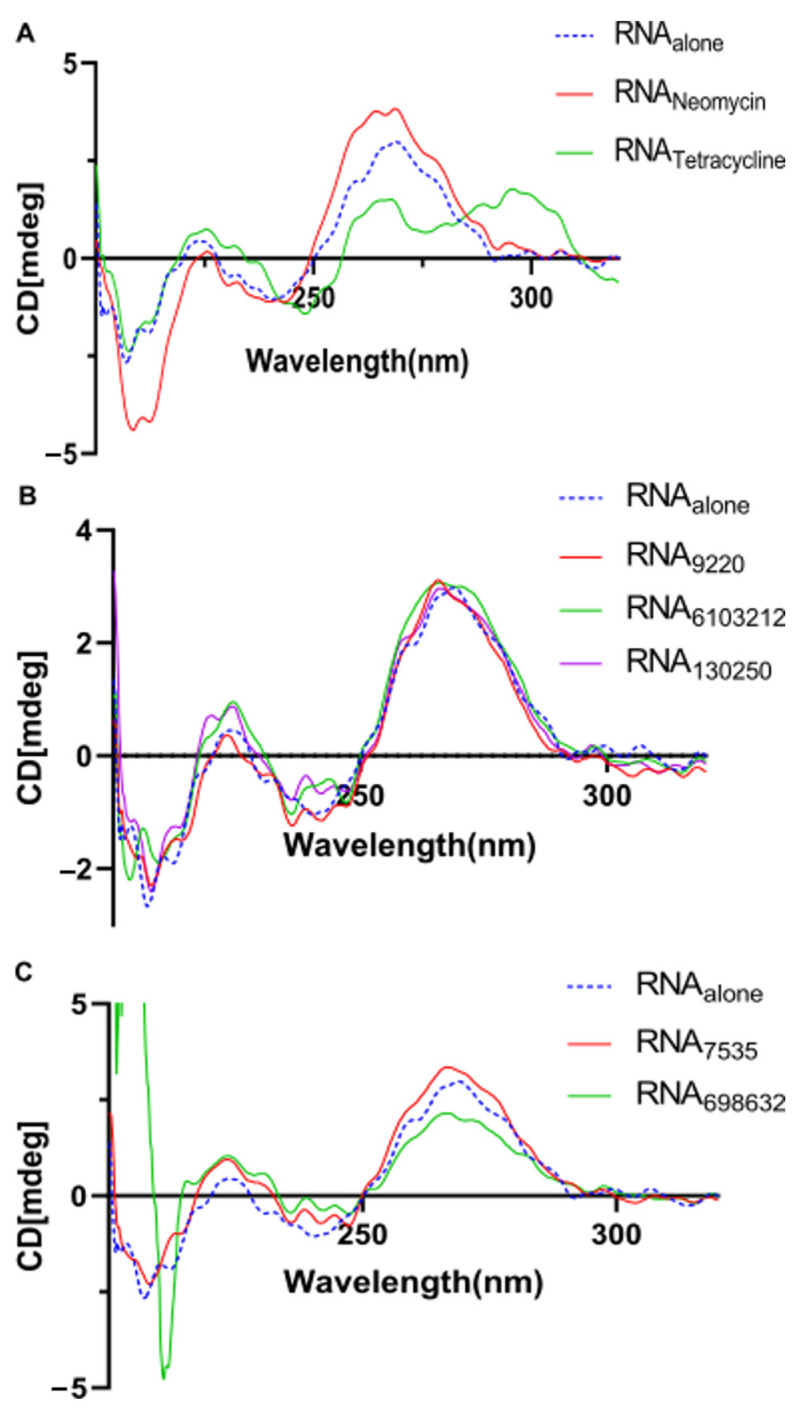
CD spectra of the U-helix RNA in complex with (**A**) known RNA binders and with (**B**,**C**) compounds that showed binding to the U-helix.

We investigated the U-helix structure using CD spectroscopy to probe the impact of the ligand binding on the U-helix helical conformation. In the absence of drugs, the U-helix gave a CD spectrum typical of the A-form RNA, including a mild and dominant positive signal at 230 and 260 nm and a negative band at 210 nm [24,25]. For the five compounds tested, there were slight changes to the signals at 210, 230, and 260 nm (Figure 12B,C). However, these changes were not large enough to alter the overall shape of the CD spectra. These results suggest that the compounds bind primarily to a double helical region of the U-helix and rules out an intercalation binding mode. Conversely, the CD spectra of the U-helix in complex with tetracycline showed dramatic changes at the 260 nm signal. This observation indicates a significant structural change upon tetracycline binding and suggests binding by intercalation. Taken together, our UV stability and CD spectral results suggest that hit binding enhances the stability of the U-helix, but this binding does not dramatically alter the conformation of the U-helix.

## 3. Discussion

The U-insertion/deletion RNA editing in the mitochondrion of the trypanosomes is essential for the bloodstream form of the parasite and is absent in mammals. These features make this editing system an attractive drug target [26]. Most editing inhibitors identified so far are directed at the proteins in the editing complexes [7,8,26,27], although some amino sugars are known to bind RNA editing substrates [20]. There are no reports of additional chemotypes that target the RNA editing substrates.

We posited that the U-helix domain of the gRNA/pre-mRNA substrate (the U-helix is considered a universal module of the guide RNA in pre-mRNA duplexes) formed at the initiation of editing could serve as a target to identify high-affinity RNA binders with the potential of inhibiting RNA editing.. We addressed this hypothesis with an integrated approach. We combined virtual screening, MD simulations, chemoinformatics, in vitro experimental ligand binding, and biophysical characterization assays. Our study identified four compounds with micromolar affinities toward the U-helix RNA target. The number of leads identified is similar to the three leads found for binding to the G-quartet RNA by using an approach similar to ours [28]. Our further characterization revealed that the compounds stabilize the U-helix and do not significantly alter the RNA’s conformation.

The reason for beginning with an in silico approach was to start from a diverse set of small molecules. If instead, we screened an extensive chemical library in the lab, this would have been an expensive campaign. Thus, the virtual screening proved to be resource- and time-efficient. Usually, due to the limitations of the docking software scoring functions, post-docking hit selection can be a challenge because false positives or similar chemo-types can be erroneously prioritized. Here, we applied an unsupervised machine learning approach that grouped structurally similar compounds into clusters. Next, we selected representatives from the clusters as hits [29,30,31]. This sampling of the clusters insured that we selected a diverse set of compounds for further testing.

As noted in previous studies about the usefulness of MD simulation as a post-docking validation tool [32], the RNA–ligand MD simulations provided further insights, some of which can be tested experimentally [33]. The amplitude of the RMSD along a molecular dynamics trajectory is a widely accepted metric that measures the deviation of the atoms of the ligands and the RNA from their starting positions. This variation provides insights into how the ligands moved during the simulation. When the RMSD values for the RNA are low, as shown in Figure 4, they suggest stability and insignificant conformational change. Conversely, weaker RNA ligand affinity is highly probable when the ligands show high RMSD values. An analysis of the RMSD values can be used to identify ligands that are likely to bind tightly.

An experimental validation of the hit compounds from the computational screens is essential in any drug discovery campaign. The microscale thermophoresis provided an efficient means to study the RNA–ligand interactions. Several reports outlined the importance of target validation through biophysical binding assays before further characterization can be performed [34,35,36]. The binding effects of the compounds identified in this study were essentially in agreement with what Leeder and others reported for aminoglycosides and other RNA-binding compounds [19,20,37,38]. Stabilizing the RNA duplex upon ligand binding suggests that these compounds can stall RNA editing by interfering with the strand separation by the RNA editing-associated helicases. The enhancement of the conformational stability and rigidity of nucleic acids is a well-reported mechanism of targeting RNA [39,40,41].

Since the discovery of cisplatin effectiveness in cancer therapy [42], numerous metal complexes or metallo-drugs have shown promise as precursors for drugs that target RNAs in cancer and other diseases, including infections caused by microbes [43]. However, these compounds are not easily used with the fast AutoDock Vina program that we used. Instead, the much slower AutoDock [17] or GemDock [44] programs were successfully used to screen metal-complex binding to proteins. However, these programs are used to screen tens of metallo-drug compounds rather than hundreds of thousands of compounds [45]. As a result of this technical limitation, we screened chemical libraries that did not include metal complexes. We left the screening of metal complexes as a future direction for our research program.

Our study outlines the discovery of new compounds beyond known aminoglycosides that target RNA substrates involved in RNA editing. The discovery and development of new compounds with improved affinities for RNA editing substrates are crucial for new drug development. The next steps include screening the compounds in RNA editing assays, and then subjecting the surviving lead compounds–RNA complexes to structural studies. The resulting experimental structures of the complexes can be used for the redesign of the compounds for an improved binding affinity. In addition, these new compounds have promise as probes of the RNA editing pathway. They can be used as tools to better understand the role of the RNA structure and dynamics during the editing process in trypanosomes. Our study’s approaches and results can serve as a basis for future efforts in the context of trypanosome RNA editing and in other well-established RNA-driven biochemical processes.

## 4. Materials and Methods

### 4.1. Compounds

Small molecule compounds identified in the virtual screening and selected in the molecular dynamics simulation were purchased as dry powders from MolPort Ltd., Riga, Latvia or donated by the Developmental Therapeutics Program (DTP) of the National Cancer Institute, Frederick, MD, USA. The compounds were dissolved in 10 mM HEPES (pH 7.5), 100 mM KCl, 0.5 mM EDTA, 2 mM MgCl_2_, and 0.1% Tween 20 at 1 mg/mL and stored at 4 °C in amber glass vials.

### 4.2. Labeled and Unlabeled RNA

A 16 nt RNA (5′-AGAGGGAAUUUUUUUU-3′) was designed to self-anneal to form a fragment of the U-helix from the gA6-14 guide RNA/gA6 mRNA editing substrate [12,46]. We designed this U-helix construct to form three contiguous G-U wobble base-pairs to mimic the G-U-rich site found in the 32 nt RNA (PDBid: 5DA6) used in the computational screen [47]. Cy5-labeled and unlabeled forms of the 16 mer U-helix and a 16 mer Watson–Crick helix (G-U base pairs replaced with G-C base pairs; 5′-AGAGGGAAUUCCCUCU-3′) were synthesized via phosphoramidite chemistry and PAGE purified by Dharmacon, Lafayette, CO, USA.

### 4.3. Virtual Screening

With the atomic-resolution crystal structure of a 32 nt U-helix (PDBid: 5da6) defined as the target RNA, the second strand was generated by applying dyad symmetry. The coordinate file was prepared for docking by removing the solvent molecules and by using AutoDock Tools (ADT) to compute the Gasteiger charges, to add polar hydrogen atoms [17] and to save the coordinates in the PDBQT file format. We carried out virtual screening of 262,000 compounds selected from the ZINC15 database [15] and from the Diversity Set of the DTP program of the NIH/NCI against the 32 nt U-helix using AutoDock Vina version 1.2.0 on the supercomputer at the Oklahoma Center for Supercomputing Education and Research, University of Oklahoma [16]. The docking grid box (25 × 25 × 25 Å) had a grid spacing of 0.375 Å and was centered on the major groove face of the G-U-rich section of the U-helix. We generated 99 poses for each compound during its docking simulation. Following virtual screening, we used the pose with the most favorable docking energy for further analysis with the molecular graphics program PyMOL [48].

### 4.4. Cheminformatic Analysis

The top 5000 compounds were ranked according to their docking score. This subset was further analyzed using RDKit [49] to calculate the molecular weight, the partition coefficient (*LogP*), and the number of hydrogen bond donors and acceptors. Compounds with favorable aqueous solubility (*LogP* < 0) were clustered by their molecular fingerprints by using the Butina clustering algorithm [18]. For molecular dynamic simulations, we selected representative compounds from each cluster that had more than five members.

### 4.5. Molecular Dynamic Simulations

We used the docked complexes of the selected compounds from the cluster analysis for MD simulations with GROMACS version 2020.3 at the High-Performance Computing Center, Oklahoma State University [50]. The simulated system was composed of the compound docked in the major groove of the 32 nt U-helix. To mimic physiological conditions, we used the solution builder in CHARMM-GUI (https://charmm-gui.org/, accessed on 30 April 2023) to construct the RNA–ligand solvent with a NaCl salt concentration of 0.15 M [37,51,52,53]. We used CHARMM36 forcefield with the TIP3P water model [54]. The RNA–ligand complexes were held at constant temperature (310 K) and pressure (1 bar) using the Nose–Hoover thermostat and the Parrinello–Rahman barostat, respectively [55].

The LINCS algorithm was used to constrain the covalent bonds containing hydrogen atoms [56], while the electrostatic and van der Waals interactions were calculated with the particle mesh Ewald method during the simulation [57]. We ran the production simulation for 50 ns with a time step of 2 fs. Following the simulation, we used the *gmx_MMPBSA* tool to calculate the binding free energy between the U-helix RNA and small molecules from the MD trajectories using the molecular mechanics/generalized Born surface area (MMGBSA) method [58,59]. Compounds with low predicted ΔG° were selected as leads for in vitro binding and melting assays.

### 4.6. Microscale Thermophoresis

We analyzed the binding affinities of the selected compounds by performing microscale thermophoresis (MST) experiments in triplicate on a Monolith NT.115 system (Nanotemper Technologies, Munich, Germany) [38,60,61]. Cy5-labelled U- and WC-helices (5′-Cy5-AGAGGGAAUU UUUUUU-3′ and 5′-Cy5-AGAGGGAAUUCCCUCU-3′) and small molecules were resuspended at a 0.5 mM concentration in 10 mM NaCacodylate pH 6.5. These solutions were added to a heat block at 95 °C for three minutes and then slow cooled over an hour to room temperature. When the temperature dropped below 40 °C, MgCl_2_ was added to a concentration of 10 mM. These RNA stocks were than used to prepare solutions with 50 nM RNA in 10 mM HEPES (pH 7.5), 100 mM KCl, 0.5 mM EDTA, 2 mM MgCl_2_, and 0.1% Tween 20. We titrated the hit compounds with a 2-fold dilution series against a 50 nM Cy5-labelled RNA. The final concentration of the compounds ranged from 800 µM to 12 nM. We incubated samples for 30 min at 25 °C and added them to capillaries (Nanotemper Technologies) for microscale thermophoresis.

The MST traces of bound and unbound RNA were subjected to temperature jump (T-Jump) analysis. The results were normalized and plotted against compound concentration. The dissociation constants for the various compounds were then determined using a single-site model to fit the binding curves. We used two known reference RNA-binding drugs (neomycin, tetracycline) as positive controls in the binding experiments. The WC-helix (no G-U wobble base pairs) served as a control on sequence-specific binding.

### 4.7. UV Melting Experiments

We assessed the thermal stability of the drug-free and drug-bound RNA in a UV melting experiment. We carried out thermodynamic measurements for nine different concentrations of the U-helix (10^−3^–10^−6^} M in binding buffer) alone or in the presence of a defined concentration of a compound on a Shimadzu UV-2600 Spectrophotometer with a thermoprogrammer (Shimadzu, Kyoto, Japan). We measured absorbance versus temperature profiles of the complexes at a wavelength of 260 nm with a heating rate of 1 °C/min from 15 °C to 90 °C. Melting temperatures were determined from the melting curves, while the thermodynamic parameters (ΔH°, ΔS°) were determined from analysis of Van’t Hoff plots [62] (ln(CT/4) versus 1/T_m_).

### 4.8. Circular Dichroism Spectroscopy

To assess the conformational changes in the secondary and tertiary RNA structures due to drug binding, we measured the CD spectra using a 0.3 mL quartz cuvette in a JASCO 715 Spectropolarimeter (JASCO, Easton, MD, USA). We used the U-helix alone or with the drug bound in the binding buffer with a final ligand concentration of 10 µM. The measurements were recorded at 25 °C in the 200–340 nm wavelength range, with a 1 nm data point interval. The CD spectra were an average of three CD measurements, with the buffer spectrum subtracted from the sample spectra [63].

## Figures and Tables

**Table 1:** Thermodynamic parameters of 16 bp U-helix in complex with compounds.

U-Helix + Drug	$T_{m}$	−ΔH°	−ΔS°
	(C)	(kJ/mol)	(eu)
U-helix alone	48.19	48.26	153.5
Neomycin	55.97	58.64	184.17
Lig7535	66.82	47.68	156.42
Lig698632	54.79	64.81	200.51
Lig6103212	72.84	33.60	109.43
Lig130250	74.63	32.43	104.74

## Data Availability

The molecular dynamics trajectories of the final five lead compounds bound to the U-helix have been deposited in Zenodo: 10.5281/zenodo.8031035.

## Abbreviations

The following abbreviations are used in this manuscript:

ADT	AutoDock Tools
CD	Circular Dichroism
CT	Strand Concentration
Cy5	Sulfo-Cyanine5
DTP	Developmental Therapeutics Program
ΔH	Enthalpy Change
EDTA	Ethylenediaminetetraacetic Acid
fs	Femtosecond
HEPES	4-1-Piperazineethanesulfonic Acid
ITC	Isothermal Titration Calorimetry
LINCS	Linear Constraint Solver for Molecular Simulations
MST	Microscale Thermophoresis
MD	Molecular Dynamics
MMGBSA	Molecular Mechanics-Generalized Born Surface Area
RMSD	Root Mean Square Deviation
nm	Nanometer
ns	Nanosecond
NCI	National Cancer Institute
pre-mRNA	Pre-Edited mRNA
ΔS	Entropy Change
SPR	Surface Plasmon Resonance
T_m_	Melting Temperature
UV	Ultra Violet
